# Sustained Complete Response to Trastuzumab Deruxtecan Beyond Treatment Discontinuation in a Heavily Pretreated HER2-Positive Breast Cancer Patient with Skin Metastases: A Case Report

**DOI:** 10.3390/reports8030126

**Published:** 2025-07-31

**Authors:** Maria Puleo, Sarah Pafumi, Martina Di Pietro, Giuseppina Rosaria Rita Ricciardi, Maria Vita Sanò

**Affiliations:** 1 Department of Medical Oncology, Humanitas Istituto Clinico Catanese, Misterbianco, 95045 Catania, Italy; maria.puleo95@gmail.com (M.P.); sarah.pafumi@humanitascatania.it (S.P.); 2 Department of Molecular Medicine, Sapienza University of Rome, 00185 Rome, Italy; 3 Department of Onco-Haematology, Papardo Hospital, 98100 Messina, Italy; martinadptr@gmail.com (M.D.P.); giusyricciardi81@hotmail.it (G.R.R.R.)

**Keywords:** long-lasting, complete response, T-DXD, ADC, advanced breast cancer, discontinuation

## Abstract

**Background and Clinical Significance**: Breast cancer is a heterogeneous disease with different spread of metastases. In particular, skin metastases are common in HER2-positive metastatic breast cancer (mBC). However, anti-HER2 therapies have shown limited activity in this context. Recently, Trastuzumab Deruxtecan (T-DXd), a novel potent anti-HER2 antibody–drug conjugate (ADC), has revolutionized the therapeutic armamentarium of HER2 mBC with unprecedented evidence of efficacy in pretreated patients. However, the activity of this drug in patients with skin involvement is largely unknown. **Case Presentation**: Here, we report a case of extensive cutaneous involvement in a heavily pretreated patient who achieved a long-lasting complete response to T-DXd, which, unexpectedly, remained sustained for more than three years following treatment discontinuation. **Conclusions**: Skin toxicity is not a common adverse event with this agent, and, as demonstrated in the present case, it might not be drug-related, and additional causes might be ruled out before treatment discontinuation. However, the possibility of discontinuing anti-Her2 treatment in a patient who has achieved a complete response could represent a field of research, potentially using liquid biopsy or other new technologies.

## 1. Introduction and Clinical Significance

The phase II DESTINY-Breast01 (DB01) trial investigated trastuzumab deruxtecan (T-DXd) at a dosage of 5.4 mg/kg in patients with HER2-positive metastatic breast cancer (mBC) yielding promising efficacy results. The confirmed objective response rate (ORR) was 62%, with 54.9% of patients achieving partial response and 7.1% achieving complete responses in the updated cumulative survival outcomes with a median follow-up of 26.5 months [1,2] The results of DESTINY-Breast01 supported the approval of T-DXd in a third-line setting by the US Food and Drug Administration in December 2019 [3,4,5]. Based on the results from DESTINY-Breast 03, the indication for T-DXd was expanded to include patients with HER2-positive mBC who had received a prior anti-HER2-based regimen in the metastatic setting or showed recurrence during/within 6 months after (neo)-adjuvant therapy [4]. The median overall survival (mOS) in DB01 was 29.1 months, demonstrating durable antitumor activity and prolonged survival with T-DXd in pretreated HER2-positive mBC patients. The findings of this study support the use of T-DXd in heavily pretreated patients with HER2-positive mBC.

Data from pivotal studies demonstrated sustained activity in most of the subgroups of patients, including the hard-to-treat population such as patients with brain metastases. However, no data are currently available on T-DXd activity in patients with skin metastases, which is a common site of disease spread, associated with substantial morbidity and worsening quality of life.

Herein, we report a long-lasting complete response with T-DXd after three years from treatment discontinuation in a heavily pretreated HER2-positive mBC patient with extensive secondary involvement of the skin.

## 2. Case Presentation

A 32-year-old premenopausal woman, with no prior history of medical conditions, was diagnosed with breast cancer. Her family history was remarkable for multiple cancer cases: one sister had breast cancer at 40 years old, another was diagnosed with colon cancer at 32 and a third had gastric cancer at 58. Due to this strong familial predisposition, she underwent a multigene panel test, including BRCA1 and BRCA2 analysis, with non-informative results.

On September, 2009, she underwent a skin-sparing mastectomy with immediate prosthetic reconstruction of the left breast with a sentinel lymph node excision. The histological examination revealed a high-grade ductal carcinoma in situ with foci of microinfiltration, the largest measuring 0.3 mm. The pathology report showed estrogen receptor (ER) and progesterone receptor (PgR) negativity, HER2 3+ positivity at immunohistochemistry, and a negative lymphatic space invasion (LSI). The pathological staging was pTmic pN0, and no adjuvant treatment was administered.

After 5 years, she underwent surgery for a recurrence in the left breast, including excision of the recurrent tumor, replacement of the left breast prosthesis, and left axillary lymphadenectomy. The pathology report revealed a micropapillary ductal carcinoma, G2, with ER and PgR negativity, Ki-67 70% and HER2 1+ at immunohistochemistry. Metastasis was detected in 1 out of 13 lymph nodes, with a pathological-stage rpT1c rpN1a. Staging workup, including whole-body CT and bone scintigraphy, showed no distant metastases. She then underwent adjuvant chemotherapy with 4 cycles of AC (doxorubicin and cyclophosphamide), followed by 12 weekly doses of paclitaxel.

In April 2016, she underwent a right nipple-sparing mastectomy with axillary lymphadenectomy due to an infiltrating ductal carcinoma. The final staging was pT2 (4.5 cm) pN3a (13 out of 22 lymph nodes involved). The tumor was ER and PgR-negative, HER2 3+ and had a Ki-67 index of 27%. She received TCH (docetaxel, carboplatin, and trastuzumab) chemotherapy, followed by one year of trastuzumab and radiotherapy to the chest wall and regional lymph nodes. Then, she started regular clinical radiological follow-up.

Two years later, a skin biopsy from a lesion at the site of the previous left mastectomy confirmed an infiltrating breast carcinoma recurrence. The tumor maintained its previous biomolecular characteristics: ER and PgR negativity, HER2 3+ positivity, and Ki-67 35%. A PET scan showed cutaneous disease in the left breast region and highly suspicious involvement of the right breast, anterior mediastinal lymph nodes, and internal mammary chain lymph nodes.

In May 2018, she started a first-line regimen with pertuzumab, trastuzumab, and docetaxel, with partial response, lasting until September 2018 when the disease progressed. She then received second-line T-DM1 for three cycles with disease progression in the skin and mediastinal lymph nodes. From February 2019 to May 2021, she started a new treatment line with capecitabine and lapatinib, achieving an initial partial response, but later experiencing cutaneous disease progression despite nodal remission.

In June 2021, considering the will of the patient to continue an oral therapy regimen, she commenced metronomic VEX therapy (vinorelbine, cyclophosphamide, and capecitabine), lasting until October 2021.

**Figure 1:**
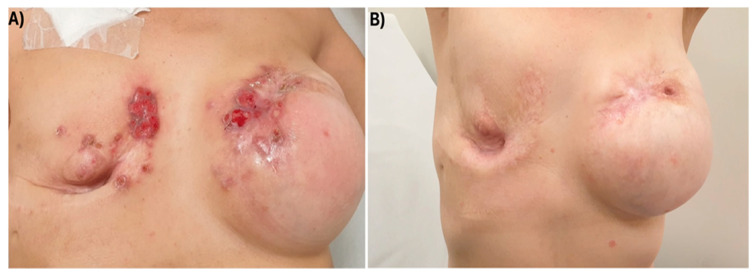
Carcinomatous mastitis extending over the skin of both reconstructed breasts before T-DXd start (**A**) and after three courses (**B**).

On November 2021, physical examination showed a carcinomatous mastitis extending over the skin of both reconstructed breasts (Figure 1), despite negative CT scan for distant metastases. She then received T-DXd from December 2021 to November 2022 (12 cycles), with a partial response after the third course and a complete remission of cutaneous metastases after 12.

**Figure 2:**
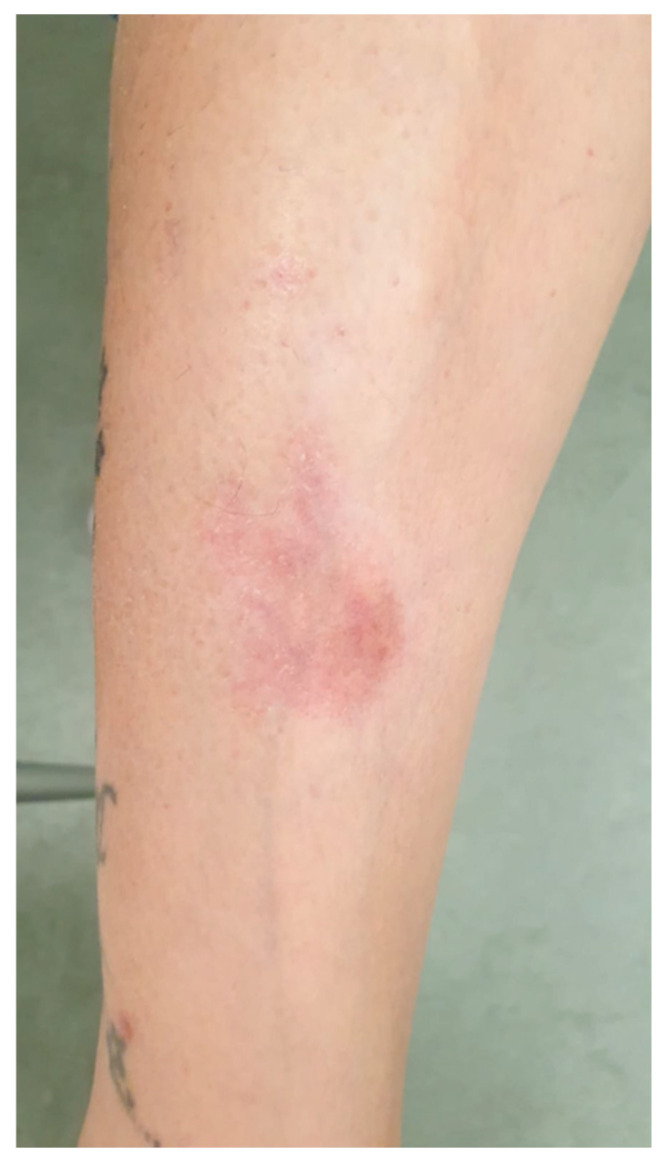
Scattered erythematous lesion on patient leg, appeared during T-DXd.

In November 2022, she developed scattered erythematous lesions on her legs, feet, buttocks, and scalp. Suspecting treatment-related cutaneous toxicity (Figure 2), T-DXd was temporarily suspended.

**Figure 3:**
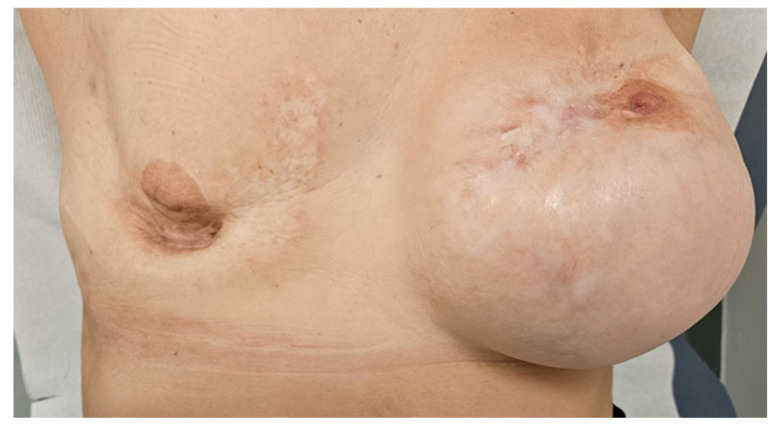
Complete remission of the carcinomatous mastitis after three years after T-DXd discontinuation.

After 30 days, she presented with widespread erythematous-desquamative plaques, particularly affecting the soles of her feet. Dermatological evaluation led to a diagnosis of psoriasis, and whole-body CT scan did not show disease recurrence. The patient decided to not resume T-DXd, and close surveillance was commenced with no disease recurrence after three years. Indeed, at the most recent follow-up visit on February 2025, over three years after discontinuing antibody–drug conjugate therapy, she continued to show a complete response (Figure 3) with no evidence of disease recurrence at CT scan and physical examination.

## 3. Discussion

Breast cancer subtypes have different metastatic spread, and cutaneous involvement represents an emerging issue [6], due to the improved survival rates yielded with the novel effective therapeutic agents [7]. Skin metastases are common in HER2-positive mBC (up to 34% of cases) [8] and might represent a sanctuary site for HER2 targeted agents [9]. Clinical presentation of skin metastases varies and might differ between breast cancer subtypes, as erythematous infiltrations seem more common in triple negative breast cancer (TNBC), while soft tissue infiltration is more frequently seen in HER2-positive and TNBC and skin ulceration in HR-positive breast cancers [8]. Novel therapeutic options for these patients are needed, as these metastases negatively impact their quality of life.

The therapeutic landscape of HER2-positive mBC has recently dramatically changed with the introduction of a novel, potent, antibody–drug conjugate targeting HER2, trastuzumab deruxtecan (T-DXd). In the pivotal phase 2 study Destiny-Breast01 (DB01), T-DXd demonstrated durable antitumor activity in pretreated HER2-positive mBC patients who had received at least two lines of anti-HER2 therapies, including trastuzumab emtansine (T-DM1), with a median of six treatment lines in the cohort of the recommended 5.4 mg/kg dose [1]. T-DXd showed an ORR of 60.9% (95% confidence interval [CI], 53.4 to 68.0), with a median PFS of 16.4 months (95% CI, 12.7 to not reached) at the first analysis (median follow-up 11.1 months) [1]. These results were confirmed at longer follow-up (median follow-up of 26.5 months) with an ORR of 62% (95% CI, 54.5% to 69.0%), a median PFS and duration of response (DoR) of 19.4 months (95% CI 14.1–25.0 months) and 18.2 months (95% CI 15.0 months; not evaluable), and a median OS of 29.1 months (95% CI 24.6–36.1 months) [3]. The promising results of the DB01 trial in HER2-positive mBC were further confirmed in two large phase 3 trials, the DB02 evaluating T-DXd versus treatment of physician’s choice [10] and the DB03 evaluating T-DXd versus T-DM1 in patients with HER2-positive mBC previously treated with trastuzumab and a taxane [3,4,5]. T-DXd demonstrated sustained and durable clinical activity even in patients with brain metastases [11]. Based on the results of these trials, T-DXd became the standard of care for previously treated HER2-positive mBC, regardless of previous treatment lines received or presence of brain metastases. However, the activity of this agent has not been reported in HER2-positive mBC metastasized to the skin. To the best of our knowledge, there are no previous reports of such a durable response following T-DXd discontinuation in HER2-positive metastatic breast cancer patients with cutaneous involvement. The long-term response seen in this heavily pretreated patient further highlights the sustained activity of this agent in this set of patients.

## 4. Conclusions

T-DXd is associated with a relatively good safety profile, with interstitial lung disease (ILD) as the most clinically relevant and potentially dangerous side effect. Skin toxicity is not a common adverse event with this agent, and, as demonstrated in the present case, it might not be drug-related and additional causes might be ruled out before treatment discontinuation. In our case, the patient’s decision not to resume therapy with T-DXd was related to quality of life. Specifically, alopecia and asthenia were the side effects that negatively impacted her quality of life. After discontinuation of the treatment, the patient once again achieved a good quality of life.

However, the possibility of discontinuing anti-Her2 treatment in patients who have achieved a complete response could represent a field of future research, potentially using liquid biopsy or other new technologies.

Liquid biopsies can detect circulating tumor DNA (ctDNA) and circulating tumor cells (CTCs) in the blood, providing real-time information on how a tumor is responding to treatment. They may help avoid unnecessary treatment in patients who are achieving a complete response and are unlikely to benefit from continued therapy. While promising, treatment decisions based on liquid biopsies—particularly regarding treatment discontinuation—are not yet part of standard clinical practice and are still being evaluated in ongoing clinical trials [12].

## Data Availability

The original contributions presented in this study are included in the article. Further inquiries can be directed to the corresponding author.

## Abbreviations

The following abbreviations are used in this manuscript:

mBC	metastatic Breast Cancer
T-DXd	Trastuzumab- Deruxtecan
DB01	Destiny Breast 01
ORR	Objective Response Rate
mOS	median Overall Survival
ER	Estrogen Receptor
PgR	Progesterone Receptor
